# CMRF: analyzing differential gene regulation in two group perturbation experiments

**DOI:** 10.1186/1471-2164-13-S2-S2

**Published:** 2012-04-12

**Authors:** Nirmalya Bandyopadhyay, Manas Somaiya, Sanjay Ranka, Tamer Kahveci

**Affiliations:** 1 Computer and Information Science and Engineering, University of Florida, Gainesville, FL 32603, USA

## Abstract

**Background:**

Microarray experiments often measure expressions of genes taken from sample tissues in the presence of external perturbations such as medication, radiation, or disease. The external perturbation can change the expressions of some genes directly or indirectly through gene interaction network. In this paper, we focus on an important class of such microarray experiments that inherently have two groups of tissue samples. When such different groups exist, the changes in expressions for some of the genes after the perturbation can be different between the two groups. It is not only important to identify the genes that respond differently across the two groups, but also to mine the reason behind this differential response. In this paper, we aim to identify the cause of this differential behavior of genes, whether because of the perturbation or due to interactions with other genes.

**Results:**

We propose a new probabilistic Bayesian method *CMRF *based on Markov Random Field to identify such genes. CMRF leverages the information about gene interactions as the prior of the model. We compare the accuracy of CMRF with SSEM and Student's t test and our old method SMRF on semi-synthetic dataset generated from microarray data. CMRF obtains high accuracy and outperforms all the other three methods. We also conduct a statistical significance test using a parametric noise based experiment to evaluate the accuracy of our method. In this experiment, CMRF generates significant regions of confidence for various parameter settings.

**Conclusions:**

In this paper, we solved the problem of finding primarily differentially regulated genes in the presence of external perturbations when the data is sampled from two groups. The probabilistic Bayesian method CMRF based on Markov Random Field incorporates dependency structure of the gene networks as the prior to the model. Experimental results on synthetic and real datasets demonstrated the superiority of CMRF compared to other simple techniques.

## Background

Microarray experiments often measure expressions of genes taken from sample tissues in the presence of external perturbations such as medication, radiation, or disease [1,2]. Typically in such experiments, gene expressions are measured before and after the application of external perturbation, and are called *control data and non-control data*, respectively. In this paper, we focus on an important class of such microarray experiments that inherently have two groups of tissue samples. Different groups in a microarray measurement can exist in many different ways. For instance, samples can be taken from members of multiple closely related species (e.g. rat versus mouse). Within the same species there can be subgroups with different phenotypes (e.g. African American versus Caucasian American). Another example is when the samples have already been through several alternative external perturbations (e.g. fasting and not fasting). When such different groups exist, it is not only important to observe overall changes in gene expression, but also to observe how different groups respond to the external perturbation. For example, Taylor et al. applied medications on 36 Caucasian American and 33 African American patients infected with Hepatitis C [3]. Gene expressions were collected before and after the medication.

In a perturbation experiment, some of the genes respond by noticeably changing their expression values between the control and non-control data. Genes that change their expressions in a statistically significant way are referred to as *differentially expressed (DE)*, while those that do not, are referred to as *equally expressed (EE) *genes. In the context of two groups, we refer to a gene that has the same state in both the groups, i.e. either DE or EE for both the groups, as *equally regulated (ER) *gene. On the contrary, if a gene is DE in one group and EE in the other, we denote it as *differentially regulated (DR)*.

**Figure 1:**
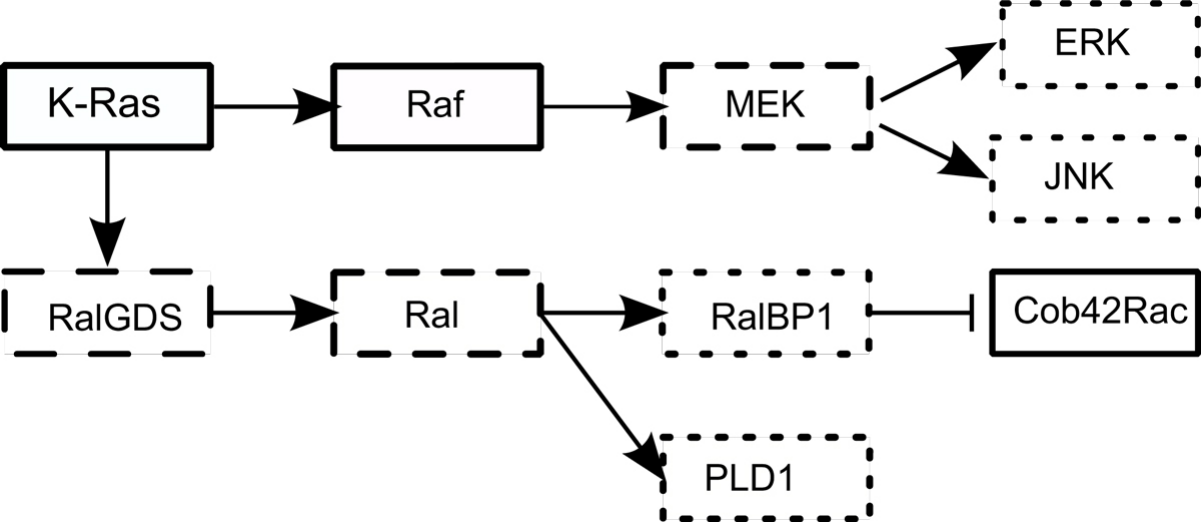
**A sample gene regulatory network**. Illustration of the impact of a hypothetical external perturbation on a small portion of the Pancreatic Cancer pathway. The pathway is taken from the KEGG database. The solid rectangles denote the genes affected directly by perturbation, the dashed rectangles indicate genes secondarily affected through the networks. The dotted rectangles denote the genes without any change in expression. → implies activation and ⊣ implies inhibition. In this figure, genes K-Ras, Raf and Cob42Roc are primarily affected and MEK, Ral and RalGDS are secondarily affected through interactions.

Genes for any organism typically interact with each other via regulatory and signaling networks. For simplicity, we will refer to them as *gene networks *for the rest of this paper. A small portion of an example gene network can be seen in Figure 1.

Once an external perturbation is applied, a gene can be DE in one of the two ways - as a direct effect of the perturbation or via interaction with other DE genes through gene networks. We denote a gene as *primarily affected *DE, if it is DE due to the external perturbation. Similarly, a gene is *secondarily affected *DE, if it is DE due to another gene in the gene network. Figure 1 shows the state of the genes in the Pancreatic Cancer pathway after a hypothetical external perturbation is applied. In this figure, genes K-Ras, Raf and Cob42Roc are primarily affected and MEK, Ral and RalGDS are secondarily affected through interactions.

Recall that for a gene to be DR, it has to be EE in one group and DE in another group. For such a gene, if it happens to be DE in one group because of the external perturbation, we call it as *primarily differentially regulated (PDR) *gene. When it is DE in one group because of the interaction with other DE genes in the gene networks, we will refer to it by *secondarily differentially regulated (SDR) *gene. *In this paper, we consider the problem of identifying the PDR genes in a given set of control and non-control gene expressions from two groups of samples*.

Existing methods to identify the primarily affected DE genes using association analysis techniques [4,5], haplo-insufficiency profiling [6-8] and chemical-genetic interaction mapping [9] are limited to applications where additional information such as fitness based assays of drug response or a library of genetic mutants is available. Bernardo et al. suggested a regression based approach named MNI that assumes that the internal genetic interactions are offset by the external perturbation [10]. It estimates gene-gene interaction coefficients from the control data and uses them to predict the target genes in the non-control data. Cosgrove et. al. proposed a method named SSEM that is similar to MNI [11]. SSEM models the effect of perturbation by an explicit change of gene expression from that of the unperturbed state.

We have also developed a method to detect the primarily and secondarily affected genes in perturbation experiments with a single data group [12]. We will call this method SMRF (single MRF) in the rest of this paper for it applies MRF on single group datasets. In that paper we developed a Bayesian probabilistic method based on Markov Random Field that leverages the information from gene networks as the prior belief of the model.

Though these methods analyze primary and secondary effects of perturbation on gene expressions, they are not directly applicable for multi-group perturbation experiments.

Several recent studies aim to identify DE genes in multiple groups of data points. maSigPro is a two stage regression based method that identifies genes that demonstrate differential gene expression profiles across multiple experimental groups [13]. Hong et al. proposed a functional hierarchical model for detecting temporally differentially expressed genes between two experimental conditions [14]. They modeled gene expressions by basis function expansion and estimate the parameters using a Monte Carlo EM algorithm. Tai et al. ranked DE genes using data from replicated microarray time course experiments, where there are multiple biological conditions [15]. They derived a multisample multivariate empirical Bayes statistic for ranking genes. Angelini et al. proposed a Bayesian method for detecting temporally DE genes between two experimental conditions [16]. Deun et al. developed a Bayesian method to find the genes that are differentially expressed in a single tissue or condition over multiple tissues or conditions [17]. All these methods identify differentially expressed genes in multiple groups*. However, none of these methods analyzed the primary and secondary effects in a two group perturbation experiment. In this paper, we develop a method to solve this problem*.

### Our approach

In this paper, we propose a new probabilistic Bayesian method CMRF to find the PDR genes in two group perturbation experiment dataset as defined above. We call this method CMRF (Comparative MRF) for it applies MRF on two groups of data for comparison purpose. Our Bayesian method incorporates information about relationship from gene networks as prior beliefs. We consider the gene network as a directed graph where each node represents a gene, and a directed edge from gene *g_i _*to gene *g_j _*represents a genetic interaction (e.g *g_i _*activates or inhibits *g_j_*). We define two genes as *neighbors *of each other if they share a directed edge. For example, in Figure 1, genes K-Ras and Raf are neighbors as K-Raf activates Ras. We also classify a neighbor as *incoming *or *outgoing*, if it is at the start or at the end of the directed edge respectively. In Figure 1, Raf is an incoming neighbor of MEK and MEK is an outgoing neighbor of Raf. When the expression level of a gene is altered, it can affect some of its outgoing neighbors. Thus, the gene expression can change due to external perturbation or because of one or more of the affected incoming neighbors.

We represent the external perturbation by a hypothetical gene (i.e. *metagene*) *g*_0 _in the gene network. We add an edge from the metagene to all the other genes because the external perturbation has the potential to affect all the other genes. So, *g*_0 _is an incoming neighbor to all the other genes. We call the resulting network the *extended gene network*. CMRF estimates the probability that a gene *g_j _*is DR due to an alteration in the activity of gene $g_{i}\left(\forall g_{i}\in G\cup \left\{g_{0}\right\},\;g_{j}\in G\right)$ if there is an edge from *g_i _*to *g_j _*in the extended network. We use a Bayesian model in our solution with the help of Markov Random Field (MRF) [18] to capture the dependency between the genes in the extended gene network. We define feature functions that encapsulate the domain knowledge available from gene networks and gene expression data. CMRF optimizes the joint posterior distribution over the random variables in the MRF using Iterated Conditional Modes (ICM) [19]. The optimization provides the state of the genes, the regulation of the genes and the probabilistic estimate of pairwise interactions between the genes including the metagene. Given this, we can rank the genes according to the data likelihood that a gene is DR because of the metagene *g*_0_, and obtain a list of possible PDR genes.

**Figure 2:**
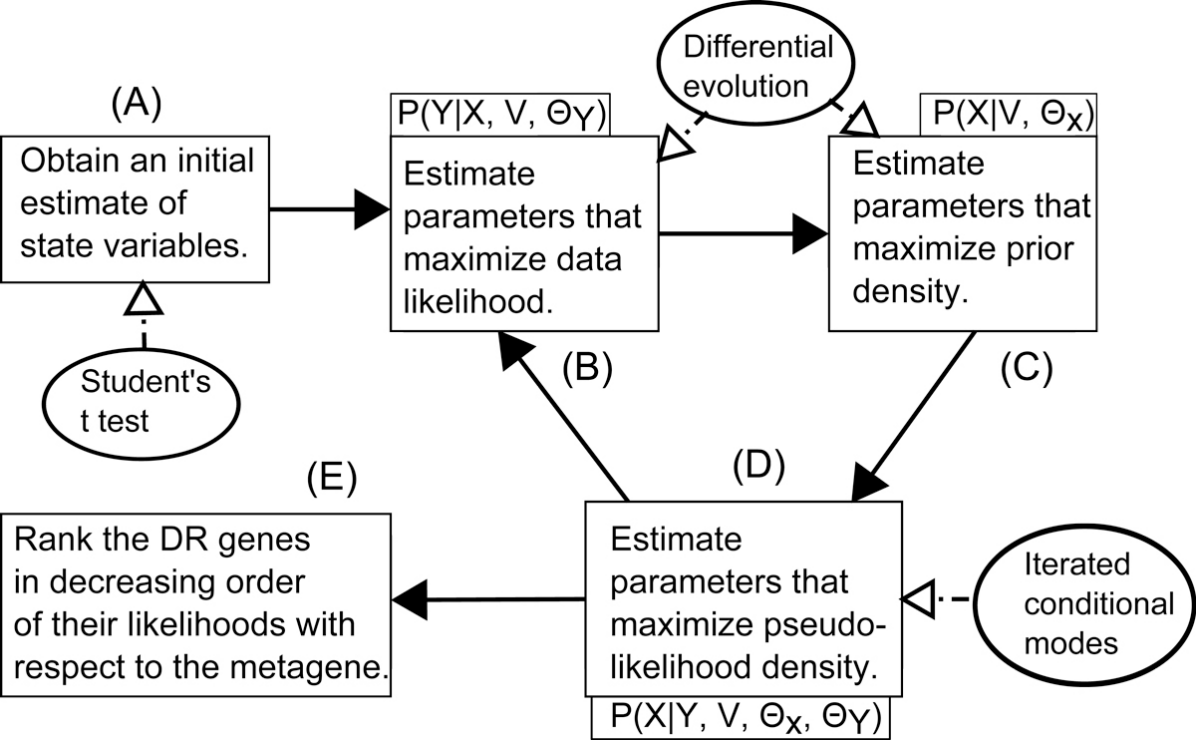
**Illustration of different components of CMRF and connectivity between them**. (A) obtains an initial estimates of state variables using Student's t test. (B) estimates parameters in a way that maximizes data likelihood. (C) estimates parameters in order to maximize prior density. Both (B) and (C) use a global optimization technique called *Differential Evolution*. (D) employs *Iterated Conditional Modes *to maximize the pseudo-likelihood. (B), (C) and (D) consist of an alternating optimization technique. These three steps (B), (C) and (D) are repeated till the algorithm meets a criteria for completion. Finally, once the optimization is complete, the DR genes are sorted in decreasing order of their likelihood with respect to the metagene *g*_0_. The genes at the top of the list are declared PDR.

Figure 2 illustrates different components of CMRF and the connectivity between them. Note that, (C) corresponds to the Bayesian prior based on MRF.

We compare the accuracy of CMRF with that of SSEM and Students t test on semi-synthetic dataset generated from microarray data in Cosgrove et al [11]. We also compare CMRF with our old method SMRF that we developed to identify the primarily affected DE genes in a single group perturbation data [12]. CMRF obtains high accuracy and outperforms all the other three methods. Also, we conduct a statistical significance test using a parametric noise based experiment to evaluate the accuracy of CMRF. In this experiment our model demonstrates reasonable confidence regions for various values of the parameters.

The rest of the paper is organized as follows. Section Results and discussion presents the results of our experiments. Section Methods describes our methods in detail. Section Conclusions concludes our discussion.

## Results and discussion

In this section we discuss the experiments we conducted to evaluate the quality of CMRF. We implemented CMRF in MATLAB and Java. We obtained the code for Differential Evolution from http://www.icsi.berkeley.edu/~storn/code.html. We compared CMRF with SSEM as SSEM is one of the most recent methods that considers identifying primarily affected genes in a perturbation experiments [11].

We obtained SSEM from http://gardnerlab.bu.edu/SSEMLasso. We executed our code on a Quad-Core AMD Opteron 2 Ghz workstation with 32 GB of memory.

### Dataset

We used four different sets of data to conduct the experiments in this paper.

• Dataset 1. The first dataset was collected by Smirnov et al. [20]. This dataset was generated using 10 Gy ionizing radiation over immortalized B cells obtained from 155 members of 15 Centre d'tude du Polymorphisme Humain (CEPH) Utah pedigrees [21]. Microarray snapshots were obtained before (at zero*th *hour) and after (at second and sixth hours) the application of radiation.

• Dataset 2. The second dataset corresponds to a drug response experiment conducted by Taylor et al [3]. Medications were applied on 36 Caucasian American and 33 African American patients infected with Hepatitis C. Gene expressions were collected before the medication was started and at 1, 2, 7, 14, 28 days after the medication was administered.

Both dataset 1 and 2 are microarray time series data with more than two time points. We adapted these two time series data two create control and non-control data suitable for our experiments. We used the data before perturbation as control data. For the non-control data we calculated the expected expression of a gene at each points after the perturbation. We selected the one with highest absolute difference from the expected expression of control data for that gene.

• Dataset 3. We created dataset 3 using dataset 1. We used the control group of dataset 1 as the control group of dataset 3. Then, we changed the expression values of some of the randomly selected genes to model the primary effect of external perturbation. From that perturbed dataset, we simulated the secondary effects using the sigmoid method described in Garg et al. [22]. We denote the parameter for primary perturbation effect by *deviation*. Deviation is the ratio of the change of expression value Δ*x *of a gene to its original expression value *x *(i.e. $derivation=\frac{\Delta x}{x}$) which is normalized between zero and one. We tuned the other parameters of the method to create a meaningful dataset as follows; *alpha *= 1, *β *= 0.01, *k_ac _*= 1.0, *k_in _*= 1, h = 0.1.

• Dataset 4. We create this dataset from dataset 1 in two steps as follows.

- Selection of genes. In order to carry out experiments on larger scale data with known PDR genes, we generated data in the presence of a hypothetical perturbation from the real datasets as follows. We first select three sets of genes. Each set consists of some primarily affected genes and a higher number of secondarily affected genes. Here, we describe how we construct each of the three sets of affected genes. We first select a random gene from the network and label it as a primarily affected DE gene. We then traverse its outgoing neighbors in a breadth first search manner. As we visit a gene during traversal, we label it as a secondarily affected DE gene with a probability of 1 − (1 − *q*)*^η^*, where *η *is the number of incoming DE neighbors. Here *q *is the probability that a gene is DE due to a DE predecessor (0.4 in our experiments). We repeat these steps to create the desired number of primarily affected genes.

After we obtain the three set of genes, we assign one set to both *D_A _*and *D_B _*groups. We assign the other two sets of genes to different groups. These two set of genes are differentially regulated as they are affected in only one group and not in the other. The three groups can contain different number of primarily and secondarily affected genes. We call these three sets of genes as primarily differentially regulated, secondarily differentially regulated and equally regulated genes.

- Generation of gene expression. Once we identify these three sets of genes in the two groups, we create control and non-control data for *D_A _*and *D_B _*over *N *samples. We use the control part of the real dataset in Smirnov et al. as the control part of our synthetic dataset in both *D_A _*and *D_B _*[20]. To generate the non-control dataset, we traverse each of the genes that participate in the gene networks. Consider a gene *g_i _*with mean and standard deviation of expression in the control dataset given by *µ_i _*and *σ_i _*respectively.

If the gene is EE we generate its non-control data points from the a normal distribution given by the parameters (*µ_i_*, $\sigma_{i}^{2}$). If the gene is DE, we use the same variance but different mean as that of the control group. For the primarily and secondarily affected genes we use *µ_i _*± *d_p _*and *µ_i _*± *d_s _*respectively, where *d_p _*>*d_s_*.

To summarize, we used the same variance in the non-control group as that in the control group. However, for an affected gene we changed the value of the mean in the non-control group from that in the control group. For a primarily affected gene we applied a higher deviation of mean than that of the secondarily affected genes.

### Regulatory networks

We collected 24,663 genetic interactions from the 105 regulatory and signaling pathways of KEGG database [23]. Overall 2,335 genes belong to at least one pathway in KEGG. In our model, we considered only the genes that take part in the gene networks.

### Comparison to other methods

Our method provides us a list of differentially regulated genes. We sort the list of those genes as follows. Consider a DR gene *g_i_*, which is DE in *D_A _*and EE in *D_B_*. We calculate the likelihood of being EE in *D_A _*and DE in *D_B _*for that gene. We can interpret this step as the probability of being DR, but in a reverse way. We could instead use the probability that the gene is DE in *D_A _*and EE in *D_B_*. However, according to our observation, the earlier metric provides a much better accuracy. We sort all the DR genes with increasing order of that likelihood.

As per our knowledge, no other method exists that differentiates between the primary and secondary effects in a two-group perturbation experiment. There exist some studies in identifying primarily affected genes in single group datasets. We compared the accuracy of CMRF to three such methods namely, SMRF, Student's t test and SSEM.

#### Experimental setup

Given an input dataset, using each of the four methods, we ranked all the genes. Highly ranked genes have higher chance of being a PDR according to each method. However, as other three methods are not tailored to solve this problem, we created separate ranking on *D_A _*and *D_B_*. Then, out of those two ranks, we created a unified rank of differentially regulated genes. We shall elaborate on this unified rank creation later. We, first, explain how we create ranks on individual groups *D_A _*and *D_B _*for other three methods.

• SMRF. We apply the SMRF to each group separately and obtain a set of differentially expressed genes. We sort the genes in decreasing order of joint likelihood with the metagene. A higher joint likelihood implies a higher chance of being primarily affected.

• SSEM. We train SSEM on the control dataset, where it learns the correlation between the genes. We test SSEM on the non-control dataset of each group, where it produces a rank for each single data point.

• Student's t test. We use the function called *ttest2 *from MATLAB. We apply it on every individual gene, where it takes control and non-control dataset as input and produces a p-value as output. We assume that the null hypothesis corresponds that the gene is EE. So a substantially lower p-value implies a higher chance of being primarily affected. We perform the test on all the genes and rank them according the increasing order of p-values.

Now we describe how we create an unified ranking of differentially regulated genes for these three methods. We denote the ranks from data group *D_A _*and *D_B _*by *R_A _*and *R_B _*respectively. The unified rank is defined by *R_U _*. We denote the number of genes in each rank to be *ω_A _*and *ω_B _*respectively. We scan both the ranks simultaneously from first position to *ω *= min(*ω_A_*, *ω_B_*). While scanning at the k*th *position, we denote the equally regulated set obtained till that position by Λ*_k _*= *R_A _*(1: *k*) ∩ *R_B _*(1: *k*). We include *R_T _*(*k*) to the unified rank *R_U _*if *R_T _*(*k*) ∉ Λ*_k_*, *T *∈ {*A*, *B*}. For SSEM we obtain a separate *R_U _*for each data point. We average the accuracies over all these ranks.

#### Results

**Figure 3:**
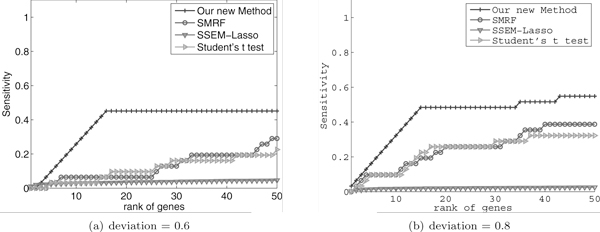
**Comparison of CMRF with three other methods**. Comparison of our method CMRF to SMRF, SSEM and t-test. The number of primarily differentially affected genes is 40. The values for deviation (maximum perturbation to the PDR genes) are 0.6 and 0.8. The figures indicate that CMRF outperforms SMRF, SSEM and t-test.

In this experiment we used dataset 3, that we have just described. To observe the accuracy of CMRF at varying degree of difficulties, we conducted experiments with four different values of *deviation*, namely, {0.5, 0.6, 0.7, 0.8}. However, we discuss only two of them in this paper (see Figure 3) since for other two parameters the results are similar. The results we discuss correspond to the cases when deviation = {0.6, 0.8}.

Figures 3(a) and 3(b) show the sensitivity of the four methods with the two deviation settings. The former one corresponds to the computationally harder case as the difference between the non-control groups of primarily and secondarily affected genes is small. As the deviation increases identifying primarily affected genes becomes easier. Form the figure, we observe that CMRF is significantly more accurate than the other three methods for all datasets consistently. It reaches almost 50% sensitivity (i.e., it can find around 15-18 primarily affected genes out of 30) in the top 50 ranked genes, when the deviation is 0.6. On the other hand, its achieves a sensitivity of 0.6 when the deviation is 0.8. We obtained similar results for other deviations, which we do not discuss here. The method in SMRF reaches to 30% and 40% accuracy, however at a slower pace. The t-test reaches around 25% and 30% sensitivity at ranking position 50 for these two cases respectively. SSEM's sensitivity is below 0.1 for all experiments even within the top 50 positions.

We believe that there are three major factors for the success of our method over the other competing methods. First, the other methods do not simultaneously handle two groups of datasets and are not able to generate an unified ranking of differentially regulated genes. CMRF encompasses both groups in a single model and probabilistically determines the PDR genes. Hence, it is more shielded against the false positives introduced during the unification of ranking. Second, CMRF can successfully incorporate the gene interactions using MRFs while others ignore this information. Finally, in real perturbation experiments, multiple genes are often primarily affected. CMRF is capable of dealing with both large and small number of primarily affected genes, while performances of other methods deteriorate as the number of primarily affected genes grows. Thus, we conclude that our method is more suitable for real perturbation experiments.

### Statistical significance experiment

The experiments in the last section enable us to compare the accuracy of CMRF with that of the other methods on synthetic datasets. We also wanted to evaluate the accuracy of CMRF on real dataset. However, we do not have any gold standard available that enlists *true *set of PDR genes. Hence, we conducted a set of statistical significance experiments to estimate the confidence of our accuracy. Specifically, we obtained the control data from a real dataset, perturbed it in a controlled way for a number of genes. We calculated the likelihood probabilities of those genes and created a distribution. We repeated this process with varying amount of perturbation. Finally, we executed CMRF on a real dataset and analyzed the result.

#### Results

We obtained the real dataset from drug response experiment conducted by Taylor et al [3], which is actually dataset 2. Apart from this real dataset, we create different versions of dataset 4 by varying *d_p _*as {0.1, 0.2, 0.3,..., 3.0}. If *d_p _*> 1.1, we set *d_s _*to 1, otherwise *d_s _*= 0.5 × *d_p_*. Thus, we have 30 synthetic datasets in total. In every dataset, we fix the number of primarily and secondarily differentially regulated genes to 50 and 172 respectively. To decide whether a gene *g_i _*is DR, when *g_i _*is DE in *D_A _*and EE in *D_B_*, we define a null-hypothesis *H*_0*i*_: *g_i _*is *DR*, *but in the reverse way, i.e. g_i _is EE in D_A _and DE in D_B_*. We calculate the likelihood of being EE in *D_A _*and DE in *D_B _*for that gene, as described. For gene *g_i_*, we denote the log likelihood of accepting *H*_0*i *_by *LL_i_*. In every dataset, we create a box plot of the 50 *LL_i _*values, as the number of DR genes in each dataset is 50. A lower value *LL_i _*indicates that *g_i _*has a higher probability of being differentially regulated.

**Figure 4:**
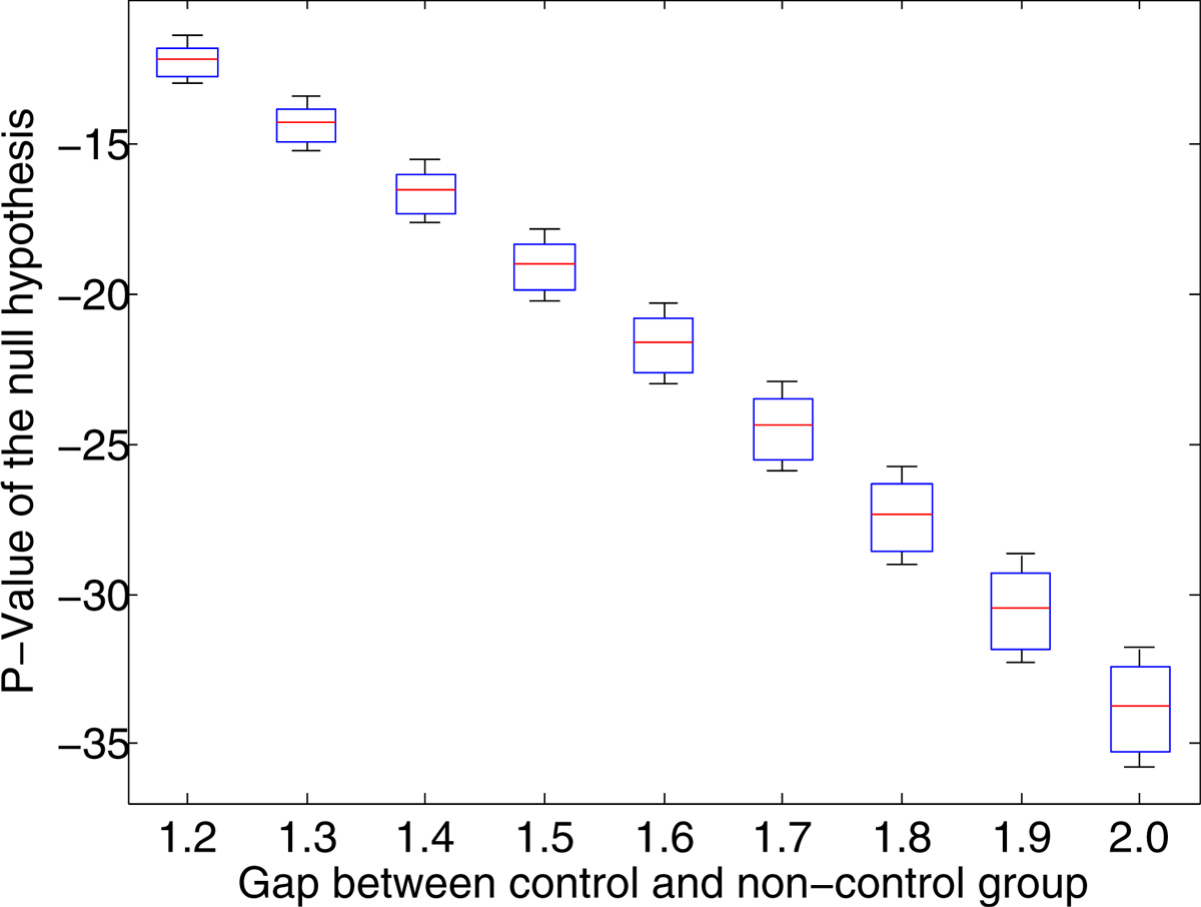
**Illustration of statistical significance test**. Illustration of the statistical significance test. Box plot demonstrates the P-value of null hypothesis of the DR genes over synthetic dataset. From the plot we clearly conclude that a higher gap between the control and non-control group of a DR gene leads to a lower P-value. The genes with a lower P-value have a higher chance of being primarily differentially regulated.

Figure 4 illustrates the statistical significance of the experiments over the datasets with *d_p _*= 1.2 to 2.0. The box plot demonstrates a relationship between the P-value and *d_p_*. A higher value of *d_p _*indicates a lower P-value and hence, a high chance of being PDR. We also observe that the variance of P-value increases with the increase of *d_p_*.

We also executed CMRF on the real datasets without any modification. Interestingly, on the real dataset from Taylor et al. [3] (dataset 2), we did not obtain any genes as differentially regulated. A careful observation concludes that when both the number of data points and the gap *d_p _*(i.e. the signal to noise ratio) is low, the coefficients *γ*_6 _and *γ*_7 _in the prior density become strong and all genes are identified as equally regulated. However, when either the number of data points or *d_p _*is significantly high, the data can overcome the prior. In the current real dataset, the number of data points is only 33 and the gaps between the control and non-control group were less than 1.2 × *σ*. As a result, CMRF identifies no differentially regulated genes in the dataset. Thus, we can conclude that either there is not much difference between the two groups in the real data, or the data does not contain enough data points, so that our model can highlight difference between the two groups.

In Figure 4 we present the results for *d_p _*= 1.2 to 2.0. Note, that for *d_p _*< 1.2 × *σ *our model did not identify any DR genes. Here also, we attribute a similar reason for not finding any DR genes as both *d_p _*and the number of data points are small. On the other hand, in Section Comparison to other methods, when we execute CMRF on synthetic datasets with 155 data points we were able to identify a substantial number of true PDR genes even with *d_p _*= 0.02 × *σ*. To substantiate our conclusion that there exists little difference between the two groups in the real dataset, we conducted a set of permutation tests. We shuffled the two original groups to create new sets of data. We repeated this process for a number of times (40 in the present experiment) and executed CMRF on each of them. For every derived dataset, CMRF did not find any DR genes. Hence, this experiment bolsters the claim that there are no DR genes in the original real data.

An interesting question can be raised is that "is there indeed no DR genes in the real dataset from Taylor et al. [3]?" Another similar question can be "will our method be able to detect DR and PDR genes from similar other real datasets?" We believe that CMRF requires a bigger dataset for DR and PDR genes to be discovered. For example, CMRF is able to identify the DR and PDR genes from the synthetic dataset that contains substantially higher number of data points than that of the real dataset. Since the difference between control and non-control groups of a DE gene is small compared to the variance of the data points, it is difficult to detect that subtle effect of perturbation with a small dataset. For a small dataset, the prior due to third hypothesis becomes strong and the two corresponding parameters γ_6 _and γ_7 _assumes extreme values. Thus the support from data is not sufficient to overcome the prior and hence, the method is not able to identify the DR and PDR genes. There are two solutions to overcome this problem. First of them is to employ a bigger dataset. With the advancement of comparatively inexpensive and high throughput technologies bigger dataset are increasingly common nowadays. From that perspective, CMRF is supposed to perform more accurately in the near future. A second option to circumvent the problem is to restrict the growth of the two parameters γ_6 _and γ_7_. If we have knowledge about the values of these two parameters, we can assign then as input to the program and refrain from estimating their values. This will enable us to employ a comparatively non-informative prior which will be easier for the data to overcome. Also, we can use specific bound over those variables while estimating them to avoid them becoming stronger.

## Conclusions

Microarray experiments often measure expressions of genes taken from sample tissues in the presence of external perturbations such as medication, radiation, or disease. Typically in such experiments, gene expressions are measured before and after the application of external perturbation.

In this paper, we solved the problem of finding primarily differentially regulated genes in the presence of external perturbations when the data is sampled from two groups. The probabilistic Bayesian method based on Markov Random Field incorporates dependency structure of the gene networks as the prior to the model. Experimental results on synthetic and real datasets demonstrated the superiority of CMRF compared to other simple techniques.

## Methods

In this section we describe different components of CMRF. Section Notation and problem formulation describes the notation and formulates the problem. Section Overview of the solution provides a high level overview of the solution. Section Computation of the prior density function describes the calculation of the prior density function of MRF. Section Approximation of the objective function discusses the definition of a tractable objective function. Section Computation of likelihood density function discusses the calculation of the likelihood function. Finally, Section Objective function optimization describes the algorithm to optimize the objective function.

### Notation and problem formulation

In this section, we describe our notation and formally define the problem. We define a Bayesian model for gene expression in a two-group perturbation experiment. We classify the random variables of the model into two different groups, namely *observed variables *and *hidden variables*. We have the values for the observed variables, while we estimate the values of the hidden variables.

#### Observed variables

We define two sets of observed variables, one for microarray gene expression data and another for the neighborhood in the extended gene network.

• Microarray data. We denote the number of genes by *M *and the number of data points in the two groups *D_A _*and *D_B _*by *N_A _*and *N_B _*respectively. We represent the set of genes with $G=\left\{g_{1},\;g_{2},\;\cdots \;,\;g_{M}\right\}$. For each gene and for each group the microarray data contains the gene expression values before and after the perturbation, i.e. control and non-control data respectively. We denote the expression value of the i*th *gene from the j*th *sample in the control data of group *D_A _*with *y_Aij_*. We represent the same for the non-control data with $y_{Aij}^{\prime }$. Thus the expression values of the gene *g_i _*for all the samples in *D_A _*for control and non-control data are $y_{Ai}=\left\{y_{Ai1},\;y_{Ai2},\;\cdots y_{AiN_{A}}\right\}$ and $y_{Ai}\prime =\left\{y_{Ai\text{1}}\prime ,\;y_{Ai\text{2}}\prime ,\;\cdots y_{AiN_{A}}\prime \right\}$ respectively. We denote all the expression values in group *D_A _*for gene *g_i _*with $Y_{Ai}\left(\text{i}.\text{e}.\;Y_{Ai}=y_{Ai}\cup {y^{\prime }}_{Ai}\right)$. We denote the collection of the gene expressions of all the genes in group *D_A _*by $Y_{A}={⋃}_{i=1}^{M}Y_{Ai}$. We define $Y_{B}$ similarly for all the genes in *D_B_*. We refer the complete gene expression data using variable $Y=Y_{A}\cup Y_{B}$.

• Neighborhood variables. We use the term $W=\left\{W_{ij}\right\}$ to indicate if two genes *g_i _*and *g_j _*are neighbors in the extended gene network. If *g_i _*is an incoming neighbor of *g_j _*(i.e. *g_j _*has an incoming edge from *g_i _*), then we set the value of *W_ij _*(1 ≤ *i*, *j *≤ *M*) to 1. It is 0 otherwise.

#### Hidden variables

We define three sets of hidden variables, These variables govern the state of genes, regulations of genes and interactions among genes respectively.

• State variables. We use $S_{A}=\left\{S_{Ai}\right\}$ and $S_{B}=\left\{S_{Bi}\right\}$, (1 ≤ *i *≤ *M*) to denote the states of the genes in group *D_A _*and *D_B_*. *S_Ai _*= 1 if *g_i _*is DE in *D_A _*and 0 if it is EE in *D_A_*. We define *S_Bi _*similarly. We assume that the metagene *g*_0 _is DE for both *D_A _*and *D_B_*. Thus, *S_A_*_0 _= *S_B_*_0 _=1.

• Regulation variables. We denote the regulation condition of gene *g_i _*with *Z_i_*. Table 1 enumerates different values of *Z_i _*for the values of *S_Ai _*and *S_Bi_*. In this formulation, the cases *Z_i _*= {2, 3} indicate that *g_i _*is DR, whereas *Z_i _*= {1, 4} indicate that *g_i _*is ER. The metagene is guaranteed to be ER, since *S_A_*_0 _= *S_B_*_0 _= 1.

• Interaction variables. In order to govern the joint regulation states of genes *g_i _*and *g_j _*we define interaction variables $X=\left\{X_{ij}\right\},\left(1\leq i,j\leq M\right)$. Mathematically, *X_ij _*= 4 × (*Z_i _*− 1) + *Z_j_*. Note that, this equation is created to maintain brevity of the mapping between the interaction variables and the regulation variables by carefully assigning different numeric constants between one and 16 to appropriate values of an interaction variable. Table 1 enumerates different values of *X_ij _*for values of *Z_i _*and *Z_j _*. Specifically, *X*_0*j *_∈ {2, 3} and *X*_0*j *_∈{1, 4} correspond to the cases where *g_j _*is DR and ER respectively because of interaction with the metagene *g*_0_.

It is easy to see that the hidden variables follow a hierarchical structure. For instance, the value of *Z_i _*depends on the values of *S_Ai _*and *S_Bi_*. Similarly, the value of *X_ij _*depends on the values of *Z_i _*and *Z_j_*. Thus, the value of the dependent variable *X_ij _*is based on the values of four independent variables *S_Ai_*, *S_Bi_*, *S_Aj _*and *S_Bj _*. Table 1 enumerates the values of *Z_i_*, *Z_j _*and *X_ij _*for different values of *S_Ai_*, *S_Bi_*, *S_Aj _*and *S_Bj _*.

**Table 1:** Enumeration of the values of *Z_i_*, *Z_j _*and *X_ij_*

*S_Ai_*	*S_Bi_*	*S_Aj_*	*S_Bj_*	*Z_i_*	*Z_j_*	*X_ij_*
DE	DE	DE	DE	1	1	1
DE	DE	DE	EE	1	2	2
DE	DE	EE	DE	1	3	3
DE	DE	EE	EE	1	4	4
DE	EE	DE	DE	2	1	5
DE	EE	DE	EE	2	2	6
DE	EE	EE	DE	2	3	7
DE	EE	EE	EE	2	4	8
EE	DE	DE	DE	3	1	9
EE	DE	DE	EE	3	2	10
EE	DE	EE	DE	3	3	11
EE	DE	EE	EE	3	4	12
EE	EE	DE	DE	4	1	13
EE	EE	DE	EE	4	2	14
EE	EE	EE	DE	4	3	15
EE	EE	EE	EE	4	4	16

Enumeration of the values of *Z_i_*, *Z_j _*and *X_ij _*for different values of *S_Ai_*, *S_Bi_*, *S_Aj _*and *S_Bj_*. The hidden variables are oriented in a hierarchical structure. For instance, the value of *Z_i _*depends on the values of *S_Ai _*and *S_Bi_*. Similarly, the value of *X_ij _*depends on the values of *Z_i _*and *Z_j_*. Thus, the value of the dependent variable *X_ij _*in turn depends on the values of four independent variables *S_Ai_*, *S_Bi_*, *S_Aj _*and *S_Bj_*.

It is worth noting that the different values that we assign to the hidden variables are categorical in nature.

#### Problem formulation

Let $G=\left\{g_{1},\;g_{2},\;\cdots \;,\;g_{M}\right\}$ denote the set of all genes. Using the definition of the neighborhood variables , we denote the collection (G,W) by  which essentially represents the gene networks. We denote the metagene by *g*_0_. Given an observed data {V,Y} we want to estimate the probabilities $p\;\left(X_{ij}=x|X-X_{ij},\;Y,\;V\right),\;x\in \left\{1,2,\;\cdots 16\right\}$.

A higher value of *p *(*X*_0*j *_= {2, 3}|·) indicates a higher probability of a gene *g_j _*being PDR. Using the estimated values of *p *(*X*_0*j *_|·), ∀*_j _*∈ {1, 2,... *M*}, we can create an ordered list of candidate PDR genes.

### Overview of the solution

This section describes a high level overview of our approach to estimate *p*(*X*_0*j*_|·), ∀*_j _*∈ {1, 2,... *M*}. One simple approach can be using a hypothesis test to find out the PDR genes in the given dataset [15]. However, the available hypothesis tests do not consider the interactions among genes in the gene network. Also, deciding on the significance of test can be a complex step. Another approach can be to use SSEM to create a rank of the potential primarily affected genes in each group separately [11]. Then we can select the top *k *genes in each group and perform a set difference to obtain the PDR genes. Though SSEM considers the correlation between the genes, it does not utilize any known information from the gene networks.

We build a Bayesian probabilistic method based on Markov Random Field where we leverage the information from gene networks as the prior belief of the model. Using Bayes theorem [24] we can write the joint probability density of interaction variables  as,

(1) $$P\left(X|Y,\;V\right)=\frac{P\left(Y|X,V,\theta_{Y}\right)P\left(X|V,\theta_{X}\right)}{\sum_{X}P\left(Y|X,V,\theta_{Y}\right)P\left(X|V,\theta_{X}\right)}$$

The first term in the numerator, $P\left(Y|X,\;V,\;\theta_{Y}\right)$, is the likelihood of the observed expression data  given the interaction variables and gene network. *θ_Y _*represents the parameters for the likelihood function. A detailed discussion of how we compute this likelihood can be found in Section Computation of likelihood density function. The second term in the numerator $P\left(X|V,\;\theta_{X}\right)$ represents this prior belief. *θ_X _*represents the parameters for the prior density function. We define a Markov Random Field (MRF) over the interaction variables  and the priors are encoded via feature functions in the MRF. Details of the priors and the associated feature functions are outlined in Section Computation of the prior density function. The denominator of Equation 1 is the normalization constant that represents the sum of the product of the likelihood and the prior over all possible assignments of interaction variables .

Given the joint probability density function outlined in Equation 1, our original problem reduces to obtaining assignments for the interaction variables  and the parameters *θ_X _*and *θ_Y _*that maximize it.

A Maximum Likelihood Estimation (MLE) of Equation 1 is practically infeasible even for a small number of genes since the number of terms in the denominator grows exponentially. Instead we use a pseudo-likelihood version of the objective function as shown in Section Approximation of the objective function. We use Iterative Conditional Modes (ICM) [19] and Differential Evolution [25] in an alternating optimization technique to maximize the pseudo-likelihood with respect to , *θ_X _*and *θ_Y_*.

After the optimization, we obtain an assignment for , *θ_X _*and *θ_Y_*. Using these assignments and the observed data, we estimate the posterior probability of all *X_ij _*variables. Using the estimated values of *p*(*X*_0*j*_|·), ∀*_j _*∈ {1, 2,... *M*}, we create an ordered list of candidate PDR genes. We elaborate on each of these steps next.

Figure 2 illustrates different portions of CMRF and the connectivity between them.

### Computation of the prior density function

In this section, we describe how we incorporate gene network as the the prior belief into our Bayesian model. From the structure and properties of gene network, we build three hypotheses and embed them into our model. We present the entire concept in three numbered subsections.

#### 1. Statement of hypotheses

Here we state the three hypotheses on the biological networks in brief.

• Hypothesis 1. In each group *D_T_*(*T *∈ {*A*, *B*}), the metagene *g*_0 _can change the state of all the other genes. Thus, all the genes can be directly affected by the external perturbation.

• Hypothesis 2. In each group *D_T_*(*T *∈ {*A*, *B*}), a gene *g_i _*can change the states of its outgoing neighbors *g_j _*in the same data group, i.e. a gene can be indirectly affected by the perturbation through genetic interactions.

• Hypothesis 3. Each gene has a high probability of being equally regulated. This follows from the observation that, often the difference between the expressions of most of the genes in two groups is small. We expect that the response of genes in these groups is very similar.

Clearly, when the data does not follow one or more of the hypotheses, the optimization function can overcome the prior belief with a strong support from the data.

#### 2. Markov Random Field construction

**Figure 5:**
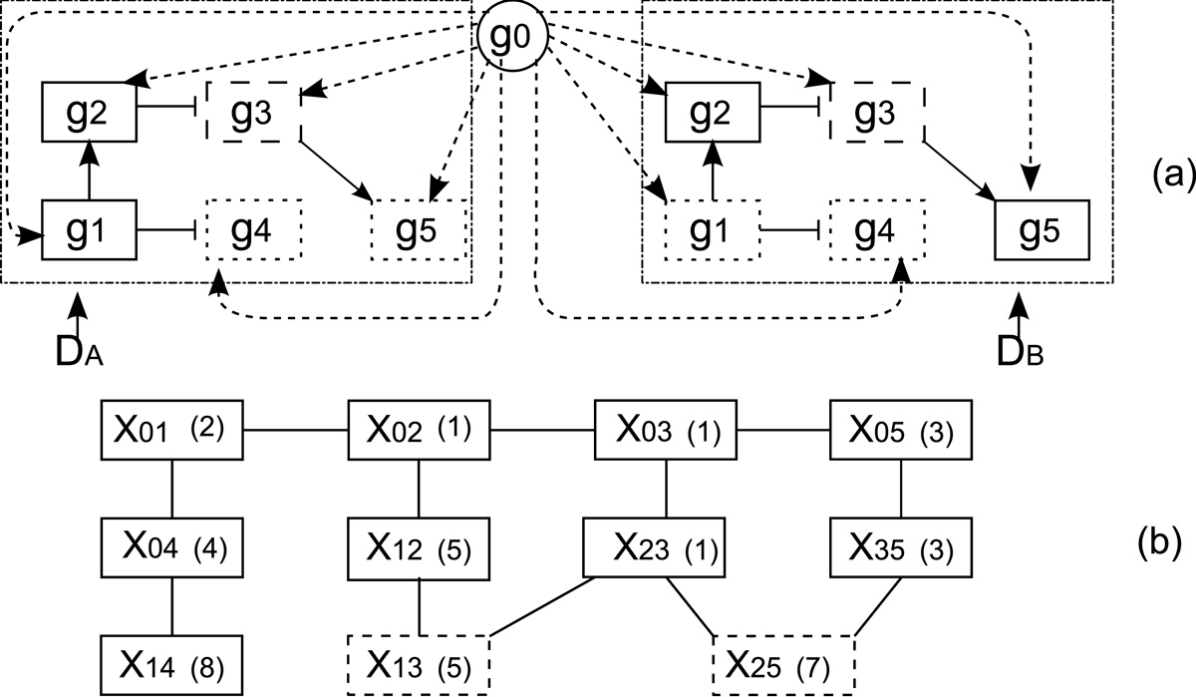
**A hypothetical gene network and corresponding Markov random graph**. (a) A small hypothetical gene network with perturbation in two datasets *D_A _*and *D_B_*. The genes in the two datasets interact through identical network, although they assume different states. The circle *g*_0 _represents the abstraction of the external perturbation. Rectangles denote genes. → implies activation and ⊣ implies inhibition. The potential effect of metagene to all other genes is indicated by dotted arrows from the metagene to all the other genes. For example, *g*_1 _is primarily affected in *D_A_*, but not affected in *D_B_*. *g*_2 _is primarily affected in both the datasets. *g*_3 _is secondarily affected in both *D_A _*and *D_B_*. (b) The Markov Random Field graph constructed based on the small hypothetical gene network in (a). The numbers in the parenthesis are the expected assignments to the variables based on the states of the genes in (a). Nodes with dotted boundaries indicate that those nodes are required for completeness of the model, however the corresponding interactions do not exist.

In order to compute the prior density function, we define a Markov Random Field (MRF) over the  variables [18]. MRF is a probabilistic model, where the state of a variable depends only on the states of its neighbors. MRF is useful to model our problem as the states of genes depend on their neighbors. Here, the MRF is an undirected graph Ψ=(X,E), where $X=\left\{X_{ij}\right\}$ variables represent the vertices of the graph (i.e. each interaction variable *X_ij _*corresponds to a vertex). We denote the set of edges with $E=\left\{\left(X_{ij},\;X_{pj}\right)|W_{pi}=W_{ij}=1\right\}\cup \left\{\left(X_{ij},\;X_{ik}\right)|W_{jk}=W_{ij}=1\right\}$. Thus, two variables in  share an edge if they share a common subscript at the same position and the two genes corresponding to the other subscript interact in the gene network. For example, in Figure 5(b), *X*_35 _and *X*_25 _are neighbors, as they share 5 (i.e. gene *g*_5_) as the second subscript and *g*_2 _and *g*_3 _interact in the gene network in Figure 5(a).

One important point to note is that, this graph does not use the state variables  or the regulation variables  to model the dependencies between the genes. Rather, it establishes those dependencies over the  variables. For example, in Figure 5(b) we draw the MRF graph corresponding to the hypothetical gene network in Figure 5(a). In the gene network, there is an edge from *g*_2 _to *g*_3_. So, *g*_2 _can potentially change the state of *g*_3_. We create an edge from *X*_12 _to *X*_13 _that corresponds to the edge from *g*_2 _to *g*_3_. As g1 is common for *X*_12 _and *X*_13_, if they assume the same value (i.e. *X*_12 _= *X*_13_), it implies that the genes *g*_2 _and *g*_3 _are in same state (i.e. *S_T_*_2 _= *S_T_*_3_,*T *∈ {*A*, *B*}). We formulate these dependency constraints using a set of unary and binary functions called *feature functions*. We discuss these feature functions next.

#### 3. Development of feature functions

We denote the neighbors of *X_ij _*in the MRF graph as $X_{ij}^{*}=\left\{X_{kj}|W_{ki}=1\right\}\cup \left\{X_{ip}|W_{jp}=1\right\}$. We define a clique over each *X_ij _*and its neighbors $X_{ij}^{*}$ by *C_ij _*provided *W_ij _*= 1. A feature function *f*(*C_ij _*) is a Boolean function defined over the clique *C_ij _*. This function evaluates to one or zero, if it is satisfied or not, respectively. We define a *potential function ψ*(*C_ij _*) corresponding to *f*(*C_ij_*) as an exponential function given by exp(*γf *(*C_ij _*)). Here γ is a coefficient associated with *f*(*C_ij_*) that represents the relevance of *f*(*C_ij_*) in the MRF. According to Hammersley-Clifford theorem, we express the joint density function of the MRF over 1471-2164-13-S2-S5 as product of potential functions defined over that MRF as, $p\;\left(X|\theta_{X}\right)=\frac{1}{\Delta }\prod_{C_{ij},W_{ij}=1}\psi \left(C_{ij}\right)$[26]. In this formulation, Δ is the normalization function $\Delta =\sum_{X}\prod_{C_{ij}}\psi (X_{ij})$. To limit the complexity of our model, we consider only cliques of size one and two.

We define seven feature functions to capture the dependencies among the variables in 1471-2164-13-S2-S5 according to the three hypotheses.

### Unary feature functions

F_1_, F2, F3. A primary component of the prior density function is modeling the frequency of *X_ij _*itself. Here, we focus on two values of *X_ij _*namely *X_ij _*= {2, 3}, since they correspond to the events that a gene *g_j _*is DR due to the metagene *g*_0_. When *X_ij _*= 2, *g_j _*is DE in *D_A _*and EE in *D_B_*. To capture this, we define a feature function *F*_1_(*X_ij_*) which returns one when *X_ij _*= 2. It returns zero otherwise. Similarly, *X_ij _*= 3 when *g_j _*is EE in *D_A _*and DE in *D_B_*. We define another feature function *F*_2_(*X_ij_*), which returns one when *X_ij _*= 3. We capture all the other values of *X_ij _*by a feature function called *F*_3_(*X_ij_*). It returns zero when *X_ij _*∈ {2, 3} and equals to one otherwise. Table 2 enumerates the the domains and ranges of *F*_1_, *F*_2 _and *F*_3_.

**Table 2:** Feature functions

*X_ij_*	*F*_1_	*F*_2_	*F*_3_	*F*_6_	*F*_7_
1	0	0	1	1	1
2	1	0	0	1	0
3	0	1	0	1	0
4	0	0	1	1	1
5	0	0	1	0	1
6	0	0	1	0	0
7	0	0	1	0	0
8	0	0	1	0	1
9	0	0	1	0	1
10	0	0	1	0	0
11	0	0	1	0	0
12	0	0	1	0	1
13	0	0	1	1	1
14	0	0	1	1	0
15	0	0	1	1	0
16	0	0	1	1	1

Enumeration of five different unary feature functions *F*_1_, *F*_2_, *F*_3_, *F*_6 _and *F*_7_.

### Binary feature functions

*F*_4_, *F*_5_. Let ϒ represent the hypothesis that in a group *D_T _*,*T *∈ {*A*, *B*} a gene *g_j _*including the metagene can change the state of one of its outgoing neighbors *g_k_*. We make a stronger hypothesis ϒ*°*that, ϒ holds simultaneously in *D_A _*and *D_B _*with high probability. Note that, this stronger hypothesis is based on the assumption that the genes in both *D_A _*and *D_B _*express in a similar fashion. This assumption is meaningful as in these two-group perturbation experiments the different groups belong to similar biological conditions [3].

ϒ*°*is encoded in 1471-2164-13-S2-S5 domain as follows. Consider four genes *g_p_*, *g_i_*, *g_j _*and *g_k_*, such that *g_p _*→ *g_i_*, *g_i _*→ *g_j _*and *g_j _*→ *g_k_*. Here → indicates that the gene on the left activates or inhibits the gene on the right. By definition, (*X_pj_*, *X_ij_*) and (*X_ij _*, *X_ik_*) are edges in the MRF. Note that the first edge corresponds to an incoming neighbor *g_p _*of *g_i_*, while the second edge corresponds to an outgoing neighbor *g_k _*of *g_j _*. We discriminate between these two sets of neighbors of *X_ij _*, as they are related to the incoming neighbors of *g_i _*and outgoing neighbors of *g_j _*respectively. It can be shown that, for the first set of edges, *X_pj _*equals to *X_ij _*if and only if (*iff*) *Z_p _*= *Z_i_*, i.e. ϒ*°*holds true. Similarly, for the second set of edges *X_ij _*equals to *X_ik _iff Z_j _*= *Z_k_*, which in tern implies that ϒ*°*is satisfied.

We define two sets of feature functions to formalize these equalities based on the incoming neighbors of *g_i _*and the outgoing neighbors of *g_j_*.

• Left external equality. We denote the incoming neighbors of *g_i _*with *In *(*g_i_*). We write a feature function *f*_4_(*X_pj _*, *X_ij_*), ∀*_p_*, *g_p _*∈ *In *(*g_i_*). *f*_4_(*X_pj _*, *X_ij_*) = 1 if *Z_i _*= *Z_p _*and *W_pi _*= *W_ij _*= 1. Otherwise, *f*_4_(*X_pj_*, *X_ij_*) = 0. We denote the summation of this function over all the incoming neighbors of *g_i _*as,

$$F_{4}\left(X_{ij}\right)=\underset{p,\;W_{ij}=1,W_{pi}=1}{\sum }f_{4}\left(X_{ij},\;X_{pj}\right).$$

• Right external equality. We denote the outgoing neighbors of *g_j _*as *Out *(*g_j_*). We define a feature function *f*_5_(*X_ik_*, *X_ij _*), ∀*_k_*, *g_k _*∈ *Out *(*g_j_*). *f*_5 _(*X_ik_*, *X_ij_*) = 1 if *S_k _*= *S_j _*and *W_jk _*= *W_ij _*= 1. Otherwise, *f*_5 _(*X_ik_*, *X_ij _*) = 0. We denote the summation of this function over all the outgoing neighbors of *g_j _*as,

$$F_{5}\left(X_{ij}\right)=\underset{k,\;W_{ij}=1,W_{jk}=1}{\sum }f_{5}\left(X_{ij},\;X_{ik}\right).$$

Tables 3 and 4 enumerate the values of *f*_4 _and *f*_5 _for different values of *X_ij_*. The missing entries in these tables correspond to the cases which can not occur during the optimization. For instance, in Table 3, a missing entry corresponds to different values of *Z_j _*in *X_ij _*and *X_pj _*which is not possible.

**Table 3:** Left external equality

									*X_pj_*								
		1	2	3	4	5	6	7	8	9	10	11	12	13	14	15	16
	1	1				0				0				0			
	2		1				0				0				0		
	3			1				0				0				0	
	4				1				0				0				0
	5	0				1				0				0			
	6		0				1				0				0		
	7			0				1				0				0	
*X_ij_*	8				0				1				0				0
	9	0				0				1				0			
	10		0				0				1				0		
	11			0				0				1				0	
	12				0				0				1				0
	13	0				0				0				1			
	14		0				0				0				1		
	15			0				0				0				1	
	16				0				0				0				1

The table enumerates the truth values for the binary feature function *left external equality *(*f*_4_). Only the possible entries are annotated with zero and one. The other entries require different values of *Z_j _*in *X_ij _*and *Xp_j_*, which is not possible. Note, that the feature function can assume one only when *X_ij _*and *X_pj _*are equal, which is in accordance with the definition of that feature function.

**Table 4:** Right external equality

									*X_ik_*								
		1	2	3	4	5	6	7	8	9	10	11	12	13	14	15	16
	1	1	0	0	0												
	2	0	1	0	0												
	3	0	0	1	0												
	4	0	0	0	1												
	5					1	0	0	0								
	6					0	1	0	0								
	7					0	0	1	0								
*X_ij_*	8					0	0	0	1								
	9									1	0	0	0				
	10									0	1	0	0				
	11									0	0	1	0				
	12									0	0	0	1				
	13													1	0	0	0
	14													0	1	0	0
	15													0	0	1	0
	16													0	0	0	1

The table enumerates the truth values for the binary feature function *right external equality *(*f*_5_). Only the possible entries are annotated with zero and one. The other entries require different values of *Z_i _*in *X_ij _*and *X_ik_*, which is not possible. Note, that the feature function can assume the value one only when *X_ij _*and *X_ik _*are equal, which is in accordance with the definition of right external equality.

For feature functions *f*_4 _and *f*_5_, *X_pj _*or *X_ik _*may not represent any interactions from the extended gene network when *W_pj _*= 0 or *W_ik _*= 0 respectively. We represent them by dotted rectangles in Figure 5(b).

### Unary feature functions

*F*_6_, *F*_7_. We introduce two unary feature functions to incorporate our last hypothesis, that all genes are ER with a high probability. We consider two genes *g_i _*and *g_j _*such that *g_i _*→ *g_j_*. This hypothesis holds true, if *g_i _*is equally regulated or *g_j _*is equally regulated.

• Left internal equality. We define this feature function to capture the events when *g_i _*is equally regulated. As, *g_j _*can assume any state, this feature function holds true for eight different values of *X_ij _*. We denote the feature function by *f*_6_(*X_ij_*, *t*) that returns one if its two arguments are equal and zero otherwise. We denote the summation of this functions over all these eight values of *X_ij _*as,

$$F_{6}\left(X_{ij}\right)=\underset{i,j,W_{ij}=1,t\in \left\{1,\cdots ,4,\;13,\cdots \;,16\right\}}{\sum }f_{6}\left(X_{ij},\;t\right).$$

• Right internal equality. We define this feature function to capture the events when *g_j _*is equally regulated. As, *g_i _*can assume any state, this feature function holds true for eight different values of *X_ij_*. We denote the feature function by *f*_7_(*X_ij_*, *t*) that returns one if its two arguments are equal and zero otherwise. We denote the summation of this functions over all these eight values of *X_ij _*as,

$$F_{7}\left(X_{ij}\right)=\underset{i,j,W_{ij}=1,t\in \left\{1,4,5,8,9,12,13,16\right\}}{\sum }f_{7}\left(X_{ij},\;t\right).$$

The last two columns of Table 2 enumerate these two internal equalities.

Based on these feature functions, we define the joint density function of 1471-2164-13-S2-S5 as,

(2) $$p\left(X|\theta_{X}\right)=\frac{1}{\Delta }exp\left(\underset{i,j,W_{ij}=1,\;k\in \;\left\{1,2,\cdots \;,7\right\}}{\sum }\gamma_{k}F_{k}\left(X_{ij}\right)\right)$$

In the above equation *γ_k_*, *k *∈ {1, 2,... 7} are the coefficients of the seven feature functions in MRF.

In the next section, we discuss how we approximate the objective function of the MRF and the data. We also describe how we formulate the posterior probability density function for *X_ij_*.

### Approximation of the objective function

A direct maximization of the objective function given by Equation 1 is intractable, as it requires evaluation of exponential number of terms in the denominator. We employ pseudo-likelihood as an established substitute to Equation 1 [27]. Pseudo-likelihood is the simple product of the conditional probability density function of the *X_ij _*variables. Geman et al. proved the consistency of the maximum pseudo-likelihood estimate [28]. The approximated objective function can be written as,

(3) $$F=\arg\underset{X}{\max}\;(\prod_{i,j}F_{ij})$$

The posterior density function *F_ij _*of *X_ij _*as,

(4) $$\begin{array}{llll} F_{ij} & =p\left(X_{ij}|X-X_{ij},\;Y,\;\theta_{X},\;\theta_{Y}\right)\; & & \;\\ & =\frac{p\left(Y_{Ai},Y_{Bi},Y_{Aj},Y_{Bj}|X_{ij},X_{ij}^{*},\theta_{Y}\right)p\left(X_{ij}|X-X_{ij},\theta_{X}\right)}{\sum_{X_{ij}\in \left\{1,\cdots \;,16\right\}}p\left(Y_{Ai},Y_{Bi},Y_{Aj},Y_{Bj}|X_{ij},X_{ij}^{*},\theta_{Y}\right)}\; & & \;\\ & \; & \end{array}$$

Derivation of ***F_ij_***.

$$\begin{array}{c} F_{ij}\\ \;=p\left(X_{ij}|X-X_{ij},\;Y,\;\theta_{X},\;\theta_{Y}\right)\\ \;=p\left(X_{ij}|X-X_{ij},\;Y_{Ai},\;Y_{Bi},\;Y_{Aj},\;Y_{Bj},\;\theta_{X},\;\theta_{Y}\right)\\ \;=\frac{p\left(Y_{Ai},Y_{Bi},Y_{Aj},Y_{Bj},X-X_{ij}-X_{ij}^{*},X_{ij},X_{ij}^{*},\theta_{X},\theta_{Y}\right)}{p\left(Y_{Ai},Y_{Bi},Y_{Aj},Y_{Bj},X-X_{ij}-X_{ij}^{*},X_{ij}^{*},\theta_{X},\theta_{Y}\right)}\\ \;=\frac{p\left(Y_{Ai},Y_{Bi},Y_{Aj},Y_{Bj},X-X_{ij}-X_{ij}^{*}|X_{ij},X_{ij}^{*},\theta_{X},\theta_{Y}\right)p\left(X_{ij},X_{ij}^{*},\theta_{X},\theta_{Y}\right)}{p\left(Y_{Ai},Y_{Bi},Y_{Aj},Y_{Bj},X-X_{ij}-X_{ij}^{*}|X_{ij}^{*},\theta_{X},\theta_{Y}\right)p\left(X_{ij}^{*},\theta_{X},\theta_{Y}\right)}\\ \;=\frac{p\left(Y_{Ai},Y_{Bi},Y_{Aj},Y_{Bj}|X_{ij},X_{ij}^{*},\theta_{X},\theta_{Y}\right)p\left(X-X_{ij}-X_{ij}^{*}|X_{ij},X_{ij}^{*},\theta_{X},\theta_{Y}\right)p\left(X_{ij},X_{ij}^{*},\theta_{X},\theta_{Y}\right)}{p\left(Y_{Ai},Y_{Bi},Y_{Aj},Y_{Bj}|X_{ij}^{*},\theta_{X},\theta_{Y}\right)p\left(X-X_{ij}-X_{ij}^{*}|X_{ij}^{*},\theta_{X},\theta_{Y}\right)p\left(X_{ij}^{*},\theta_{X},\theta_{Y}\right)}\\ \;=\frac{p\left(Y_{Ai},Y_{Bi},Y_{Aj},Y_{Bj}|X_{ij},X_{ij}^{*},\theta_{X},\theta_{Y}\right)p\left(X-X_{ij}-X_{ij}^{*},X_{ij},X_{ij}^{*},\theta_{X},\theta_{Y}\right)}{p\left(Y_{Ai},Y_{Bi},Y_{Aj},Y_{Bj}|X_{ij}^{*},\theta_{X},\theta_{Y}\right)p\left(X-X_{ij}-X_{ij}^{*},X_{ij}^{*},\theta_{X},\theta_{Y}\right)}\\ \;=\frac{p\left(Y_{Ai},Y_{Bi},Y_{Aj},Y_{Bj}|X_{ij},X_{ij}^{*},\theta_{X},\theta_{Y}\right)p\left(X,\theta_{X},\theta_{Y}\right)}{p\left(Y_{Ai},Y_{Bi},Y_{Aj},Y_{Bj}|X_{ij}^{*},\theta_{X},\theta_{Y}\right)p\left(X-X_{ij},\theta_{X},\theta_{Y}\right)}\\ \;=\frac{p\left(Y_{Ai},Y_{Bi},Y_{Aj},Y_{Bj}|X_{ij},X_{ij}^{*},\theta_{X},\theta_{Y}\right)p\left(X_{ij}|X-X_{ij},\theta_{X},\theta_{Y}\right)p\left(X-X_{ij},\theta_{X},\theta_{Y}\right)}{p\left(Y_{Ai},Y_{Bi},Y_{Aj},Y_{Bj}|X_{ij}^{*},\theta_{X},\theta_{Y}\right)p\left(X-X_{ij},\theta_{X},\theta_{Y}\right)}\\ \;=\frac{p\left(Y_{Ai},Y_{Bi},Y_{Aj},Y_{Bj}|X_{ij},X_{ij}^{*},\theta_{X},\theta_{Y}\right)p\left(X_{ij}|X-X_{ij},\theta_{X},\theta_{Y}\right)}{p\left(Y_{Ai},Y_{Bi},Y_{Aj},Y_{Bj}|X_{ij}^{*},\theta_{X},\theta_{Y}\right)}\\ \;=\frac{p\left(Y_{Ai},Y_{Bi},Y_{Aj},Y_{Bj},X_{ij},X_{ij}^{*},\theta_{X},\theta_{Y}\right)p\left(X_{ij}^{*},\theta_{X},\theta_{Y}\right)p\left(X_{ij}|X-X_{ij},\theta_{X},\theta_{Y}\right)}{p\left(X_{ij},X_{ij}^{*},\theta_{X},\theta_{Y}\right)p\left(Y_{Ai},Y_{Bi},Y_{Aj},Y_{Bj},X_{ij}^{*},\theta_{X},\theta_{Y}\right)}\\ \;=\frac{p\left(Y_{Ai},Y_{Bi},Y_{Aj},Y_{Bj},\theta_{X}|X_{ij},X_{ij}^{*},\theta_{Y}\right)p\left(X_{ij},X_{ij}^{*},\theta_{Y}\right)p\left(X_{ij}^{*},\theta_{X},\theta_{Y}\right)p\left(X_{ij}|X-X_{ij},\theta_{X},\theta_{Y}\right)}{p\left(X_{ij},X_{ij}^{*},\theta_{X},\theta_{Y}\right)p\left(Y_{Ai},Y_{Bi},Y_{Aj},Y_{Bj},\theta_{X}|X_{ij}^{*},\theta_{Y}\right)p\left(X_{ij}^{*},\theta_{Y}\right)}\\ \;=\frac{p\left(Y_{Ai},Y_{Bi},Y_{Aj},Y_{Bj}|X_{ij},X_{ij}^{*},\theta_{Y}\right)p\left(\theta_{X}|X_{ij},X_{ij}^{*},\theta_{Y}\right)p\left(X_{ij},X_{ij}^{*},\theta_{Y}\right)p\left(X_{ij}^{*},\theta_{X},\theta_{Y}\right)p\left(X_{ij}|X-X_{ij},\theta_{X},\theta_{Y}\right)}{p\left(Y_{Ai},Y_{Bi},Y_{Aj},Y_{Bj}|X_{ij}^{*},\theta_{Y}\right)p\left(X_{ij},X_{ij}^{*},\theta_{X},\theta_{Y}\right)p\left(\theta_{X}|X_{ij}^{*},\theta_{Y}\right)p\left(X_{ij}^{*},\theta_{Y}\right)}\\ \;=\frac{p\left(Y_{Ai},Y_{Bi},Y_{Aj},Y_{Bj}|X_{ij},X_{ij}^{*},\theta_{Y}\right)p\left(X_{ij},X_{ij}^{*},\theta_{X},\theta_{Y}\right)p\left(X_{ij}^{*},\theta_{X},\theta_{Y}\right)p\left(X_{ij}|X-X_{ij},\theta_{X},\theta_{Y}\right)}{p\left(Y_{Ai},Y_{Bi},Y_{Aj},Y_{Bj}|X_{ij}^{*},\theta_{Y}\right)p\left(X_{ij},X_{ij}^{*},\theta_{X},\theta_{Y}\right)p\left(X_{ij}^{*},\theta_{X},\theta_{Y}\right)}\\ \;=\frac{p\left(Y_{Ai},Y_{Bi},Y_{Aj},Y_{Bj}|X_{ij},X_{ij}^{*},\theta_{Y}\right)p\left(X_{ij}|X-X_{ij},\theta_{X},\theta_{Y}\right)}{p\left(Y_{Ai},Y_{Bi},Y_{Aj},Y_{Bj}|X_{ij}^{*},\theta_{Y}\right)}\\ \;=\frac{p\left(Y_{Ai},Y_{Bi},Y_{Aj},Y_{Bj}|X_{ij},X_{ij}^{*},\theta_{Y}\right)p\left(X_{ij}|X-X_{ij},\theta_{X}\right)}{p\left(Y_{Ai},Y_{Bi},Y_{Aj},Y_{Bj}|X_{ij}^{*},\theta_{Y}\right)}\\ \;=\frac{p\left(Y_{Ai},Y_{Bi},Y_{Aj},Y_{Bj}|X_{ij},X_{ij}^{*},\theta_{Y}\right)p\left(X_{ij}|X-X_{ij},\theta_{X}\right)}{\sum_{X_{ij}\in \left\{12,3,\cdots 16\right\}}p\left(Y_{Ai},Y_{Bi},Y_{Aj},Y_{Bj}|X_{ij},X_{ij}^{*},\theta_{Y}\right)} \end{array}$$

In step 2 of the derivation, we substitute  by *Y_Ai_*, *Y_Bi_*, *Y_Aj _*and *Y_Bj _*as *X_ij _*is independent of all *Y_Ck _*such that *k *≠ {*i*, *j*} and *C *≠ {*A*, *B*}. Also, in the 15*th *step we assume that *X_ij _*is independent of *θ_Y _*given $X-X_{ij}$ and *θ_X_*.    □

Derivation of $p\left(X_{ij}|X-X_{ij},\theta_{X}\right)$, *W_ij _*= 1.

$$\begin{array}{c} p\;\left(X_{ij}|X-X_{ij},\;\theta_{X}\right)\\ \;=\frac{p\left(X,\theta_{X}\right)}{P\left(X-X_{ij},\theta_{X}\right)}\\ \;=\frac{p\left(X,\theta_{X}\right)}{\sum_{X_{{}_{ij}}\in \left\{1,2,3\;\cdots \;16\right\}}P\left(X-X_{ij},X_{ij},\theta_{X}\right)}\\ \;=\frac{A\left(X_{ij}\right).B\left(ij\right)}{\sum_{t=\left\{1,2,3,\;\cdots \;16\right\}}A\left(t\right)\cdot B\left(ij\right)} \end{array}$$

*A *(*X_ij_*) is $exp\left(\sum_{k\in \left\{1,2,\cdots \;,7\right\}}\gamma_{k}F_{k}\left(X_{ij}\right)\right)$ and *B*(*ij*) is given by $exp\left(\sum_{m,n,ij\neq mn,k\in \left\{1,2,\cdots \;,7\right\}}\gamma_{k}F_{k}\left(X_{mn}\right)\right)$. Here, we denote the prior density parameters {*γ*_1_, *γ*_2_,...·*γ*_7_} by *θ_X_*. Canceling *B*(*ij*) from numerator and denominator the density function simplifies to,

$$\begin{array}{c} p\left(X_{ij}|X-X_{ij},\;\theta_{X}\right)\\ =\frac{exp\left(\sum_{k\in \left\{1,2,\cdots \;,7\right\}}\gamma_{k}F_{k}\left(X_{ij}\right)\right)}{\sum_{t=\left\{1.2.3,\cdots 16\right\}}exp\left(\sum_{k\in \left\{1,2,\cdots \;,7\right\}}\gamma_{k}F_{k}\left(X_{ij}=t\right)\right)} \end{array}$$

□

There are two different terms in objective function of Equation 4. $p\left(X_{ij}|X-X_{ij},\theta_{X}\right)$ stands for the conditional prior density function of *X_ij _*which we just have derived from using Bayes rule. In the next section, we discuss the likelihood function $p\left(Y_{Ai},\;Y_{Bi},\;Y_{Aj},\;Y_{Bj}|X_{ij},\;X_{ij}^{*},\;\theta_{Y}\right)$.

### Computation of likelihood density function

In this section, we describe how we derive the likelihood function in three numbered subsections. Here, we assume that gene expressions in a group follow a normal distribution, We can rewrite the derivations if gene expressions follow some other distribution.

#### 1. Likelihood for a single gene

Consider a set of measurements for a gene *g_i _*that follows a single Gaussian distribution by **z_i _**= {*z_i_*_1_, *z_i_*_2_,..., *z_iN_*}. We denote the latent mean of **z_i _**as *µ *and the standard deviation as *σ*. As different genes can have different average expressions, we assume that *µ *follows a genome wise distribution with mean *µ*_0 _and standard deviation *τ *[29]. Thus, for **z_i_**, the likelihood for the data points in that group is given by,

(5) $$\begin{array}{llll} L\;\left(z|\mu_{0},\;\sigma^{2},\;\tau^{2}\right) & =\int \left[\overset{n}{\underset{i=1}{\prod }}N\left(z_{i}|\mu ,\;\sigma^{2}\right)\right]N\left(\mu |\mu_{0},\;\tau^{2}\right)d\mu \; & & \;\\ & =\frac{\sigma }{{\left(\sqrt{2\pi }\sigma \right)}^{n}\sqrt{n\tau^{2}+\sigma^{2}}}exp\left(-\frac{\sum_{i}z_{i}^{2}}{2\sigma^{2}}-\frac{\mu_{0}^{2}}{2\tau^{2}}\right).\; & & \;\\ & \;\;exp\left(\frac{\frac{\tau^{2}n^{2}{\bar{z}}^{2}}{\sigma^{2}}+\frac{\sigma^{2}\mu_{0}^{2}}{\tau^{2}}+2n\bar{z}\mu_{0}}{2\left(n\tau^{2}+\sigma^{2}\right)}\right)\; & & \;\\ & \; & \end{array}$$

The derivation of Equation 5 can be obtained from Demichelis et al [30]. If a gene is DE, its expression measurements in control and non-control groups follow different distributions [29]. On the other hand, for equally expressed genes, all the measurements in both groups share the same mean. The likelihood function for a DE gene *g_i _*in group *D_T _*,*T *∈ {*A*, *B*} is given by,

(6) $$L_{{\text{T}}_{{}_{\text{DE}}}}\left(g_{i}\right)=L\left(y_{i}|\mu_{0},\;\sigma^{2},\;\tau^{2}\right)L\left({y^{\prime }}_{i}|\mu_{0},\;\sigma^{2},\;\tau^{2}\right)$$

Similarly, for EE genes it is given by,

(7) $$L_{{\text{T}}_{{}_{\text{EE}}}}\left(g_{i}\right)=L\left(y_{i}\cup {y^{\prime }}_{i}|\mu_{0},\;\sigma^{2},\;\tau^{2}\right)$$

For instance, the likelihood of a gene to be DE in group *D_A _*is given by $L_{A_{{}_{DE}}}\left(g_{i}\right)$.

#### 2. Likelihood for a regulation variable

As for a gene *g_i_*, the regulation variable *Z_i _*can assume four different values from 1 to 4, the equations of the likelihood that a gene is DR or ER also take four different forms given by,

$$L_{\text{Z}}\left(g_{i}\right)=\left\{\begin{array}{c} L_{{\text{A}}_{{}_{\text{DE}}}}\left(g_{i}\right)\;L_{{\text{B}}_{{}_{\text{DE}}}}\left(g_{i}\right),\;\text{if}\;Z_{i}=1.\\ L_{{\text{A}}_{{}_{\text{DE}}}}\left(g_{i}\right)\;L_{{\text{B}}_{{}_{\text{EE}}}}\left(g_{i}\right),\;\text{if}\;Z_{i}=2\\ L_{{\text{A}}_{{}_{\text{EE}}}}\left(g_{i}\right)\;L_{{\text{B}}_{\text{DE}}}\left(g_{i}\right),\;\text{if}\;Z_{i}=3\\ L_{{\text{A}}_{{}_{\text{EE}}}}\left(g_{i}\right)\;L_{{\text{B}}_{{}_{\text{EE}}}}\left(g_{i}\right),\;\text{if}\;Z_{i}=4 \end{array}\right)$$

#### 2. Likelihood for an interaction variable

We have 16 different forms for the likelihood of the *X_ij _*due to its 16 different values. However, here, we shall derive only for *X_ij _*= 1, as for the other values of *X_ij _*we have a similar derivation.

(8) $$\begin{array}{c} p\left(Y_{Ai},\;Y_{Bi},\;Y_{Aj},\;Y_{Bj}|X_{ij}=1,\;X_{ij}^{*},\;\theta_{Y}\right)\\ \;=\underset{\tau_{i},\tau_{j}\in \;\left\{1,\cdots \;,4\right\}}{\sum }p\left(Y_{Ai},\;Y_{Bi},\;Y_{Aj},\;Y_{Bj}|Z_{i}=\tau_{i},\;Z_{j}=\tau_{j},\;\theta_{Y}\right).\\ \;\;\;p\left(Z_{i}=\tau_{i},\;Z_{j}=\tau_{i},\;\theta_{Y}|X_{ij}=1,\;X_{ij}^{*},\;\theta_{Y}\right) \end{array}$$

From the definition of $X_{ij},\;p\left(Z_{i}=\tau_{i},\;Z_{j}=\tau_{i},\;\theta_{Y}|X_{ij}=1,\;X_{ij}^{*},\;\theta_{Y}\right)$ equals to 1 when *Z_i _*= 1 and *Z_j _*= 1. Its value is zero for all other values of *Z_i _*and *Z_j_*. So, continuing from the last step of Equation 8,

(9) $$\begin{array}{c} p\left(Y_{Ai},\;Y_{Bi},\;Y_{Aj},\;Y_{Bj}|X_{ij}=1,\;X_{ij}^{*},\;\theta_{Y}\right)\\ \;=p\left(Y_{Ai},\;Y_{Bi},\;Y_{Aj},\;Y_{Bj}|Z_{i}=1,\;Z_{j}=1,\;\theta_{Y}\right)\\ \;=p\left(Y_{Ai},\;Y_{Bi}|Z_{i}=1,\;Z_{j}=1,\;\theta_{Y}\right).\\ \;\;\;p\left(Y_{Aj},\;Y_{Bj}|Z_{i}=1,\;Z_{j}=1,\;\theta_{Y}\right)\\ \;=p\left(Y_{Ai},\;Y_{Bi}|Z_{i}=1,\;\theta_{Y}\right)p\left(Y_{Aj},\;Y_{Bj}|Z_{j}=1,\;\theta_{Y}\right)\\ \;=L_{\text{Z}}\left(g_{i}\right)L_{\text{Z}}\left(g_{j}\right) \end{array}$$

In a similar way, we can derive the likelihood functions for all the 16 different values of *X_ij _*variables. A special case arises when *g_i _*is the metagene, i.e. *g*_0_. We assume that $L_{{\text{T}}_{{}_{\text{DE}}}}\left(g_{0}\right)=1$ and $L_{{\text{T}}_{\text{EE}}}\left(g_{0}\right)=0,\;T\in \left\{A,\;B\right\}$. Thus, the likelihood of the metagene given *Z*_0 _= 1 equals to 1. Its value is zero otherwise.

### Objective function optimization

So far, we have described how we compute the posterior density function. The final challenge is to find the values of the hidden variables that maximize the objective function (Equation 3). We develop an iterative algorithm to address this challenge.

In our model we have three different sets of parameters. The nodes of the MRF given by 1471-2164-13-S2-S5 consist of one set. Other two sets are the parameters of conditional probability density function of *X_ij _*and likelihood function of observed data given by *θ_X _*= {*γ*_1_,... *γ*_7_} and *θ_Y _*= {*µ*_0_, *σ*, *τ*), respectively. In each iteration, we first estimate *θ_X _*and *θ_Y _*based on the estimated value of 1471-2164-13-S2-S5 in the previous iteration. Next, based on the estimated parameters, we estimate 1471-2164-13-S2-S5 that maximize the objective function in Equation 3.

The likelihood function is non-convex in terms of the parameters *θ_Y _*= {*µ*_0_, *σ*, *τ*). Also, the conditional density is non-convex in terms of *θ_X _*= {*γ*_1_,... *γ*_7_}. We use a global optimization method called differential evolution to optimize both of them [25]. To optimize the objective function in Equation 3, we employ the ICM algorithm described by Besag [19]. Briefly, our iterative algorithm works as follows.

1. Obtain an initial estimate of  variables. In our implementation we use student's t-test assuming the data follows normal distribution. We use 5% confidence interval for this purpose.

2. Estimate parameters θ*_Y _*that maximizes the data likelihood function given by,

$$\text{arg}\underset{\theta_{Y}}{\text{max}}\underset{X_{ij},W_{ij}=1}{\prod }p\left(Y_{Ai},\;Y_{Bi},\;Y_{Aj},\;Y_{Bj}|X_{ij},\;X_{ij}^{*},\;\theta_{Y}\right)$$

We implement this step using Differential Evolution, which is similar to the genetic algorithm.

3. Calculate an estimate of the parameters *θ_X _*that maximizes the conditional prior density function by,

$$\text{arg}\underset{\theta_{X}}{\text{max}}\underset{X_{{}_{ij}},W_{{}_{ij}}=1}{\prod }p\left(X_{ij}|X-\left\{X_{ij}\right\},\;\theta_{X}\right)$$

We also implement this step using Differential Evolution.

4. Carry out a single cycle of ICM using the current estimate of , *θ_X _*and *θ_Y_*. For all *S_i _*, maximize $\prod_{X_{mn}}p\left(X_{mn}|X-X_{mn},\;Y,\;\theta_{X},\;\theta_{Y}\right)$ when $X_{mn}\in \left\{X_{rt}|r=i\;\text{or}\;t=i,\;W_{rt}=1\right\}$.

5. Go to step 2 for a fixed number of cycles or until 1471-2164-13-S2-S5 converges to a certain predefined value.

We optimize the objective function in terms of the *S_i _*(1 ≤ *i *≤ *M*) variables instead of *X_ij _*variables. Specifically, in step 4, we go over all the *S_i _*variables, and optimize *F_ij _*function (given by Equation 4) for only those *X_ij _*variables that are impacted by the change of *S_i_*. Figure 2 illustrates different components of CMRF and the connectivity between them.

The optimization procedure is guaranteed to converge since in every iteration the value of the objective function increases. We continue the iterative process, until the changes in estimates of the parameters between two consecutive iterations reach below a certain cutoff level.

## Competing interests

The authors declare that they have no competing interests.

## Authors' contributions

NB conceived the study, analyzed the data, implemented the methods, supplied the analysis tools, designed the experiments, performed the experiments and wrote the paper. MS conceived the study and participated in writing the paper. SR conceived the study, designed the experiments and participated in writing the paper. TK conceived the study, designed the experiments and participated in writing the paper.
